# The latest research progress of ultrasound technology in the diagnosis of neonatal necrotizing enterocolitis: A review

**DOI:** 10.1097/MD.0000000000049235

**Published:** 2026-06-05

**Authors:** Jing Wang, Huiyu Lu, Yao Li, Zemin Zhang, Zilong Yu

**Affiliations:** a Department of Ultrasound, The Second People’s Hospital of Weifang, Weifang, Shandong, China; b Department of Campus Hospital, Weihai Technician College, Weihai, Shandong, China; c Department of Obstetrics and Gynecology, Weifang Maternal and Child Health Hospital, Weifang, Shandong, China; d Department of Pediatric Surgery, Weifang People’s Hospital, Weifang, China; e Department of Clinical Lab, Weifang Ruiqing Hospital, Weifang, Shandong, China.

**Keywords:** diagnosis, neonatal necrotizing enterocolitis, ultrasound

## Abstract

Necrotizing enterocolitis (NEC) is a common gastrointestinal disease in newborns, with insidious onset, rapid progression, and severe complications. Although abdominal X-ray examination is the standard method for diagnosing NEC, it has the disadvantages of difficulty in early diagnosis and a low positive detection rate. With the development of ultrasound technology, it has advantages such as non-radiation and dynamic, repeatable examination, and is increasingly being applied in the diagnosis of NEC.

## 1. Introduction

Necrotizing enterocolitis (NEC) is a critical disease in the neonatal period, which tends to occur in preterm infants and children with low or very low birth weight.^[1–3]^ The mortality rate of NEC can reach 20% to 40%, and even those who survive are often accompanied by sequelae such as intestinal adhesion, intestinal stenosis, short bowel syndrome, and abnormal nervous system development.^[4–6]^ The specific pathogenesis of NEC is still unclear. At present, intestinal ischemia and hypoxia, dysbacteriosis, and mucosal pro-inflammatory and anti-inflammatory imbalance are considered to be the main pathogenesis of NEC.^[7]^In terms of diagnosis, abdominal X-ray photography (AXR) has long been considered the radiological gold standard for the diagnosis and determination of NEC severity, as well as the tracking of disease progression.^[8]^However, AXR has the disadvantages of low sensitivity, repeated radiation, and difficulty in early diagnosis. In recent years, with the development of abdominal intestinal ultrasound technology, it has been widely used in the diagnosis of NEC due to its advantages of flexible operation, noninvasive nature, and dynamic real-time detection.^[9,10]^ Therefore, this article reviews the application of abdominal ultrasound technology in the diagnosis of NEC.

## 2. Historical progress of ultrasound in the diagnosis and treatment of NEC

In 1964, Berdon^[11]^ was the first to apply ultrasound technology to the diagnosis of NEC. Since then, a number of studies have shown that ultrasound can provide a more valuable reference for the diagnosis and treatment of NEC than AXR. In 1984, Kodroff et al.^[12]^ proposed that ultrasound had more advantages than X-ray in the early prediction of intestinal necrosis and perforation in NEC. In 1992, Bomelburg et al.^[13]^ conducted a 3-year follow-up study on 27 children with NEC symptoms, and the results showed that ultrasound was superior to X-ray in showing portal vein gas. In 2005, color Doppler ultrasound (CDFI) was first used to assess the blood supply of the intestinal wall.^[14]^ The abdomen was divided into 4 quadrants; each quadrant was examined by gray scale and color Doppler ultrasound, and the blood perfusion was divided into 3 states: normal, increased, and decreased. The increased blood perfusion showed a “Y-shaped” pattern, circular pattern and “zebra” pattern. Color Doppler ultrasound was more sensitive than X-ray in the diagnosis of intestinal necrosis in NEC. In 2007, Silva et al^[15]^ believed that ultrasound could make up for the deficiency of X-ray and could also predict the outcome of NEC well, providing reference information for clinical decision-making. In a 2018 questionnaire conducted in Germany, 58% indicated that their hospital used ultrasound for NEC.^[9]^ This indicates that the value of abdominal ultrasound techniques in the diagnosis of NEC has received increasing attention.

## 3. Ultrasound in the diagnosis of suspected or early NEC

NEC is a critical gastrointestinal disease in the neonatal period. Early symptoms are often atypical. At the same time, early diagnosis and treatment are key factors that determine the prognosis of children. Therefore, the early diagnosis of NEC is particularly important. For children with suspected or early onset NEC, ultrasound detection has more advantages than abdominal X-ray, which can detect pathological manifestations before the appearance of X-ray features and accurately determine the progress of intestinal changes.^[15,16]^ In the early stage of NEC, the intestinal inflammatory mediators lead to slow peristalsis, increased blood perfusion of the intestinal wall, edema and thickening of the intestinal wall, and even the formation of submucosal microbubbles. The intestine of normal newborns usually moves at least 10 times per minute.^[17]^In children with NEC, intestinal peristalsis is reduced or absent (<10/min) due to pathological factors.^[17]^Wall perfusion of the normal intestine can be assessed by color Doppler, typically detecting 1 to 9 colored signal points per square meter. At a color signal point, the Doppler velocity is 0.029 to 0.11 m/s, indicating normal physiological intestinal wall perfusion. However, some literature^[18]^ believes that when the color blood flow signal increased to 0.09 m/s, the blood perfusion of the intestinal wall was considered to increase. The normal intestinal wall thickness of newborns varies between 1 and 2.6 mm.^[19]^ In the initial stages of NEC, inflammation and edema are manifested by an uneven increase in bowel wall thickness (>2.5 to 2.7 mm).^[13,17,19–21]^This is followed by disease progression with ischemia and necrosis, and thinning of the bowel wall (usually <1 mm). < ,^8,17,19]^ In 1 study analysis, newborns with thin bowel walls had a higher risk of requiring surgery or dying than those with thick bowel walls.^[22]^ The results of Shiou et al^[23]^ showed that the sensitivity and specificity of ultrasound in the diagnosis of NEC were higher than those of other imaging methods, and ultrasound could detect bubbles with a diameter of <1 mm,  <  it could detect intestinal wall gas or portal vein gas that could not be detected by X-ray in the early stage of NEC. It has been found that unnecessary antibiotic use and duration can be significantly reduced in infants without gas on ultrasound, who would previously have received a full course of antibiotics. These findings suggest that ultrasound aids clinical decision-making by confirming or ruling out NEC based on the presence or absence of pneumatosis.^[24]^This allows effective measures to be taken before more severe symptoms occur and potentially adverse outcomes can be avoided.^[25]^

## 4. Study on ultrasound in the diagnosis of middle and late NEC

**Figure 1.**
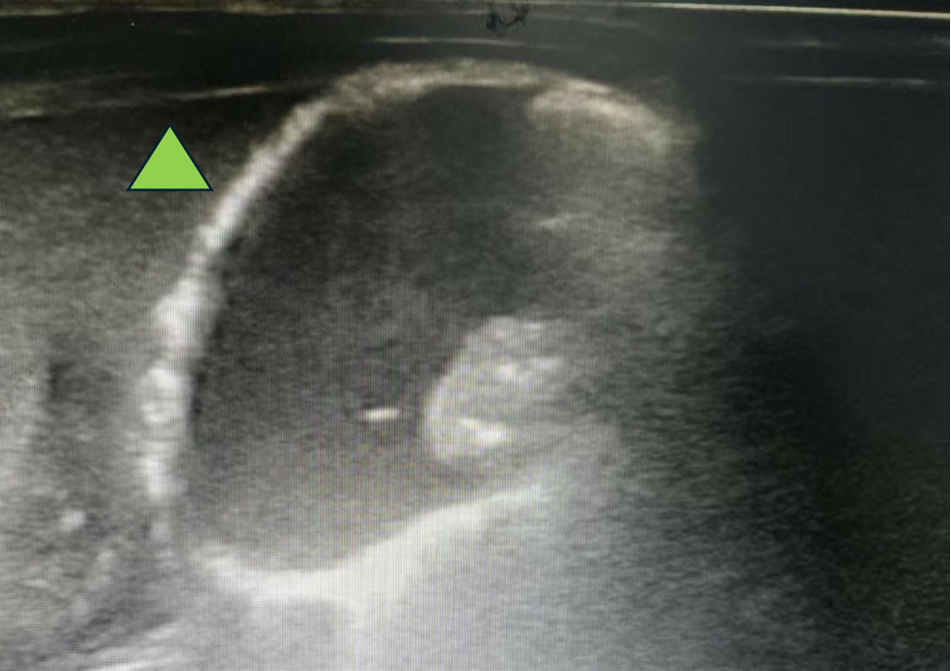
Strong echogenic gas images can be seen within the intestinal wall.

**Figure 2.**
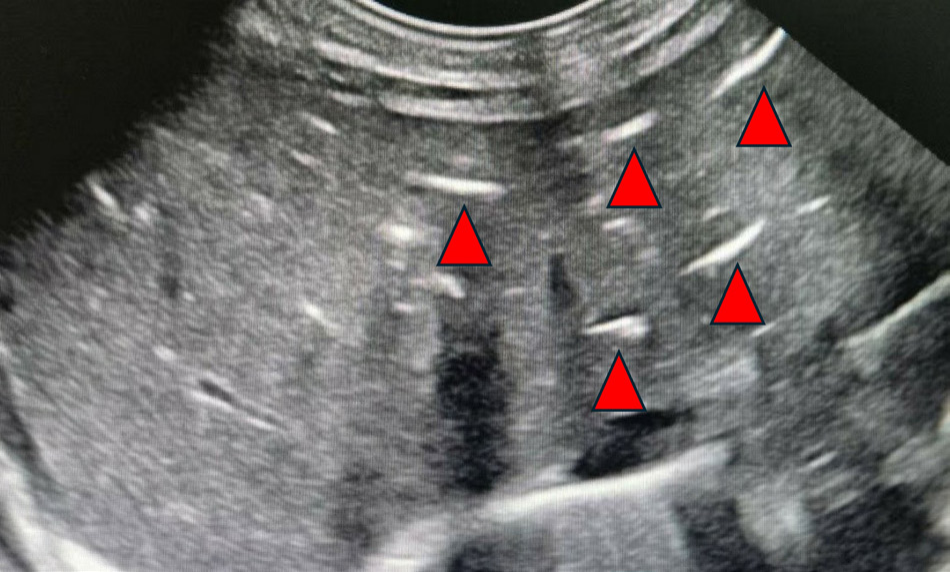
Strong echogenic gas images can be observed in the portal vein of the liver.

It is well known that the characteristic findings of NEC are pneumatosis intestinalis (Fig. 1) and portal venous pneumatosis (Fig. 2). However, AXR has limited ability to identify intestinal wall gas, portal vein gas, or even small amounts of free gas in the abdominal cavity.^[19,26,27]^ There is increasing evidence that real-time ultrasound can better detect intestinal wall air and portal vein gas than X-rays,^[1,13,15,17,28–30]^ especially in advanced NEC.^[31]^ In addition, ultrasound can directly evaluate the intestinal wall and detect the thickening or thinning of the intestinal wall, the degree of edema, blood perfusion (Table 1), and the fluid in the abdominal cavity.^[31]^ In the late stage of NEC, gastrointestinal perforation, free abdominal gas, and complex peritoneal effusion (when an echogenic substance or septum is found, peritoneal fluid is considered complex), intestinal wall thinning (<1 mm),^[1]^ and poor or even disappearance of blood perfusion are often accompanied. Free gas is the only accepted absolute indication for surgery to date,^[32,33]^ but it may not be seen in a large number of occult intestinal perforations in neonates.^[34,35]^ Early diagnosis of necrotic bowel is the key to improving the outcome of these affected neonates. However, abdominal ultrasound is very sensitive in detecting small amounts of free air and can confirm perforation as early as possible.^[16,36–38]^ Other findings^[13,16,17,30,31,39]^ showed that the presence of complex fluid in the abdominal cavity combined with a lack of Doppler blood flow signals highly suggested intestinal gangrene and required surgical intervention. The critical point of surgical treatment of NEC is the threshold of intestinal perforation, but this time is often difficult to grasp. Ultrasound is particularly helpful in the assessment of intra-abdominal conditions, such as pneumoperitoneum, focal effusion, and complex free fluid, which are considered to be high-risk indicators of perforation, and it has been reported in the literature that the presence of these factors usually requires surgical intervention.^[13,18,22,29,40]^Li Yan et al^[41]^ analyzed the ultrasonographic manifestations of 68 NEC children and combined the 3 indicators of weakened intestinal peristalsis, peritoneal effusion depth > 17.9 mm, and poor peritoneal effusion sound transmission to predict the need for surgical treatment, with a sensitivity of 95.2% and a specificity of 85.1%.

**Table 1:** Normal intestinal ultrasound and intestinal-related changes in children with NEC.

	Normal children patients	NEC
Intestinal peristalsis	>10/min	<10/min
Intestinal wall thickness	1–2.6 mm	Early stage >2.5–2.7mm
		Later period <1 mm
Small bubbles in the intestinal wall	None	Yes
Blood perfusion	Normal	Increase in the early stage

NEC = necrotizing enterocolitis.

May LA et al^[42]^ mentioned the concept of simplified ultrasound in the literature and pointed out that it is helpful in the application of accurate and timely diagnosis of NEC in the pediatric emergency department. Typically, we performed a complete NEC ultrasound protocol. In the assessment of portal gas and abdominal findings, all 4 quadrants of the bowel are evaluated.^[1,13,22,40,43]^The simplified ultrasound protocol for NEC only assesses portal vein gas and evaluates the abdominal cavity in all 4 quadrants. “In many cases, detailed bowel assessment is not feasible or impractical for more severe neonates in the emergency department, and neonates may not tolerate prolonged ultrasound procedures to complete bowel assessment.” On the contrary, it is very important to quickly determine the presence or absence of pneumoperitoneum to ascertain the presence or absence of intestinal perforation. They showed a mean reduction of 8.4 minutes in the duration of the simplified ultrasound examination (*P* < .001). In addition, the sensitivity, specificity, and negative predictive value of simplified NEC ultrasound were 100% (95%CI 60–100%), 95% (95%CI 86–98%), and 100% (95%CI 94–100%), respectively. The data suggest that a simplified ultrasound protocol is sufficient to identify patients at high risk of bowel perforation for prompt surgical treatment. The high-risk detection rate was 15% in the simplified ultrasonography group and 16% in the full ultrasonography group, indicating that there was no significant difference in the detection of bowel perforations. Moreover, there were no false negatives in the simplified NEC ultrasound group, and in no case did follow-up ultrasound lead to a decision to change clinical surgery.

Le Cacheux C et al^[44]^ presented 4 ultrasound features rarely mentioned in the literature for diagnosing NEC. These features include thickening of the mesentery accompanied by complex echoes, higher echoes of intestinal contents within the cavity than the intestinal wall, liver, or spleen, abnormal thickening and congestion of the abdominal wall, and blurred and unclear edges of the intestinal wall. The occurrence of these 4 ultrasound features often indicates a poor prognosis for treatment. Hu et al^[45]^ found that the diagnostic effect of high-frequency ultrasound for NEC is similar to that of X-ray contrast enema. In addition, high-frequency ultrasound does not require contrast agents or ionizing radiation and can be reused. These characteristics are particularly important for infants. Not only can stenosis be seen, but also the proximal intestine and small intestine can be observed. The ultrasound diagnostic features of colonic stenosis after NEC include narrowed colonic cavity, segmental ring-like thickening of the intestinal wall, low-echo fibrous exudation, and adhesions around colonic stenosis. This indicates that ultrasound detection also has obvious advantages in detecting colonic stenosis as a complication of NEC.

## 5. Application of other new ultrasound techniques in the diagnosis of NEC

At present, contrast-enhanced ultrasound (CEUS) has been used to evaluate intestinal diseases in preterm infants. CEUS can help conventional abdominal ultrasound evaluate the intestinal perfusion pattern of NEC and improve the diagnostic accuracy and prognostic value of color Doppler ultrasound. CEUS can also be used to evaluate the perfusion of the intestinal wall and determine the prognosis. In the initial stage of injury, the signal of CEUS shows hyperenhancement, and when the intestinal wall is ischemic, it shows hypoenhancement or no enhancement. The advantages of CEUS include a higher sensitivity to reduced blood flow compared with Doppler abdominal ultrasound.^[46,47]^ However, further clinical experience and prospective studies are needed to understand the pathophysiological and prognostic significance of dynamic perfusion patterns.

Microvascular Superb microvascular imaging (SMI) is an emerging microvascular imaging technology in recent years. It can be divided into 2 modes: color superb microvascular imaging (cSMI) and gray scale SMI (mSMI). The former is a color blood flow imaging mode, which is more suitable for observing objects with a small sampling area, and the latter is a monochromatic mode that can improve sensitivity by filtering out background information. SMI has a higher recognition degree for low-velocity blood flow and fine blood vessels and uses a high frame rate, high image resolution, and relatively few motion artifacts. High-frequency ultrasound combined with cSMI examination is safe and noninvasive, can be carried out dynamically and continuously, and reduces motion artifacts. cSMI technology can more accurately reflect the blood supply of the intestinal wall. It can improve CDFI blood flow grading and accurately judge whether there is ischemia or necrosis of the intestinal wall. SMI is a new ultrasound image processing technology that uses advanced clutter suppression technology to extract flow signals of tiny blood vessels and display them at a high frame rate.^[48]^ As a noninvasive examination, SMI can show the vascular structure of the lesion site in detail without the use of contrast agents and other invasive operations.

## 6. Summary and prospect

With the development of ultrasound technology, ultrasound is increasingly used in the diagnosis and evaluation of NEC. Although there are numerous reports on the application of ultrasound techniques in the diagnosis of NEC at all stages of its pathogenesis, it lacks clear staging and diagnostic criteria. In addition, ultrasonography is an operator-dependent technique with certain interindividual differences, and skilled and experienced operators are needed to make an accurate diagnosis. Therefore, it is necessary to establish standardized ultrasound diagnostic criteria for the diagnosis of NEC and its progression.

## Author contributions

**Data curation:** Jing Wang, Huiyu Lu.

**Methodology:** Yao Li.

**Writing – original draft:** Zemin Zhang.

**Writing – review & editing:** Zilong Yu.
